# Effect of Qmix 2in1, chlorhexidine gluconate, and ethylenediaminetetraacetic acid on postoperative pain after root canal treatment: A double-blind randomized clinical trial

**DOI:** 10.34172/joddd.2022.011

**Published:** 2022-05-29

**Authors:** Selen İnce Yusufoğlu, Keziban Olcay

**Affiliations:** ^1^Department of Endodontics, Faculty of Dentistry, Ankara Yıldırım Beyazıt University, Ankara, Turkey; ^2^Department of Endodontics, Istanbul University-Cerrahpaşa, Istanbul, Turkey

**Keywords:** Chlorhexidine gluconate, Ethylenediaminetetraacetic acid, Endodontic treatment, Postoperative pain, Qmix

## Abstract

**Background.** This study aimed to investigate the effects of different final irrigation solutions on postoperative pain following root canal treatment.

**Methods.** Eighty-nine nonvital premolar and molar teeth with periapical lesions (PAI: 3‒4) without any clinical symptoms were included. The patients were randomly assigned to three groups according to the final irrigation solutions used: G1: 2 mL of Qmix (n=29), G2: 2 mL of 17% ethylenediaminetetraacetic acid (EDTA) (n=30), and G3: 2 mL of 2% chlorhexidine gluconate (CHX) (n=30). All the patients were prescribed 100 mg of flurbiprofen to use as needed for pain. The patients were asked to rate their pain status according to the verbal rating scale at 12, 24, 48, and 72 hours, and one week. The data were analyzed using Pearson’s chi-squared test, Fisher’s exact test, and chi-squared analysis with Monte Carlo simulation. The significance level was set at *P*≤0.05.

**Results.** No significant differences were observed in postoperative pain rates at 12, 48, and 72 hours and one week (*P*>0.05). However, in the Qmix group, a significantly lower pain level was observed at 24 hours with EDTA and CHX (*P*=0.019). The rate of mild pain in the EDTA group at 72 hours (18.8%) was significantly higher in premolar teeth than in molar teeth (*P*=0.012). The moderate pain level in the EDTA group at 12 hours was significantly higher in those>60 years of age (*P*=0.008).

**Conclusion.** The use of Qmix as an irrigation solution resulted in lower postoperative pain levels at 24 hours compared to other solutions. Therefore, Qmix can be considered a proper final irrigation solution in endodontic treatment regarding postoperative pain.

## Introduction

 The main goal of root canal treatment is to hermetically obturate the root canal system with sufficient biomechanical material to heal periradicular tissues while minimizing patient discomfort.^1^ Postoperative pain following root canal treatment is a common but undesirable condition. A systematic review showed that the incidence of postoperative pain following root canal treatment ranged from 3% to 58%.^2^The instrumentation process depends on the clinician’s experience^3^ and factors such as instrument type, instrument kinematics, etc. Therefore, postoperative pain is considered multifactorial. Debris extrusion to the periapical tissues might occur during root canal preparation.^4^ Debris, which includes infected dentin and bacteria, is a primary cause of postoperative pain.^5^Irrigation solutions and medicaments used during root canal preparation procedures might cause irritation and chemomechanical injury of periradicular tissues, resulting in postoperative pain.^2,6^Sodium hypochlorite (NaOCl), ethylenediaminetetraacetic acid (EDTA), and chlorhexidine gluconate (CHX) solutions are recommended during instrumentation to optimize root canal disinfection.^7^ No irrigation solution has both sufficient antibacterial efficacy and the ability to dissolve organic and inorganic tissues, which are the main features expected from an irrigation solution.^8^For these reasons, NaOCl, EDTA, or CHX are used routinely as the final irrigation solutions in root canal treatment.^9^Many studies have reported that removing the smear layer is important to achieve effective adhesion between root canal filling and dentin.^7,10^It has been reported that the smear layer can be completely removed by NaOCl and EDTA in combination.^11,12^Qmix 2in1 (Qmix, Dentsply Tulsa, Maillefer, Ballaigues, Switzerland) contains EDTA, CHX, a nonspecific detergent, and water. It is an endodontic irrigation solution that is as effective as 17% EDTA in removing the smear layer.^13,14^In addition, many studies have shown Qmix to be effective in destroying microorganisms^15^ and recommended its use as the final irrigation solution after following irrigation with NaOCl.^14^

 To the best of our knowledge, there are few studies in the literature to have investigated the effect of Qmix as the final irrigation solution in endodontic treatment on postoperative pain.^16^This study aimed to evaluate the effects of EDTA, CHX, and Qmix as the final irrigation solutions in endodontic treatment on postoperative pain. The primary outcome measure of the study was to assess if different final irrigation solutions influence the occurrence of postoperative pain. The secondary outcome measure of the study was to compare postoperative pain levels between the groups by tooth type.

## Materials and Methods

###  Patient selection

 The method and design of this prospective, randomized, double-blind clinical trial were approved by the Ethics Board of the University (No: 929). The study protocol was published and registered at www.clinicaltrials.gov (identifier: NCT04310254). Before inclusion, all the patients were informed of the study protocol, aims, complications, and possible risks, and written informed consent was obtained from each patient. Patients who met the inclusion criteria were treated by a single endodontist (SIY) from the Faculty of Dentistry, Department of Endodontics, between October 2019 and February 2020.

###  Sample size determination 

**Figure 1:**
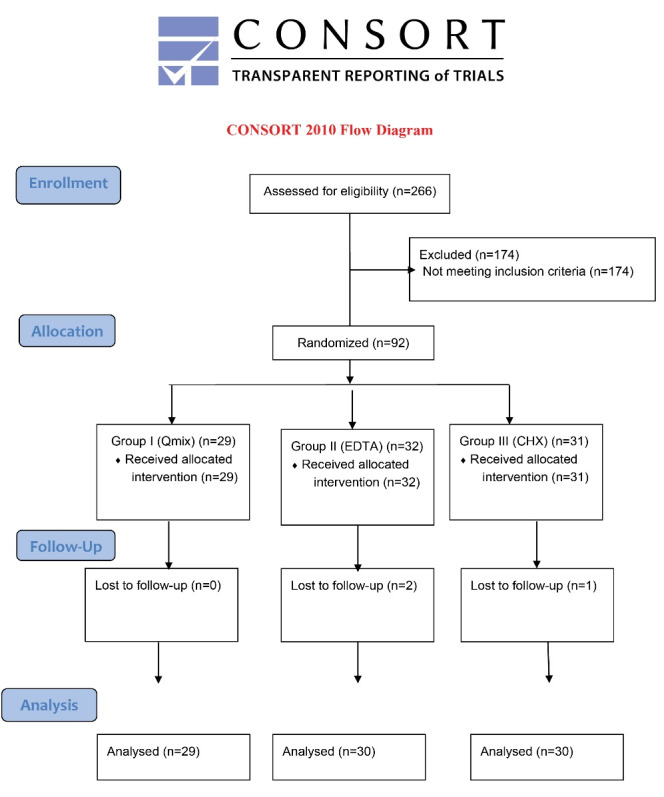
Consolidated Standards of Reporting Trials (CONSORT) flowchart

 A sample size estimation was determined using G*Power software (Version 3.1.9.7) to achieve a minimum 20% decrease in postoperative pain at 80% study power with an effect size of 35% and a significance level of 5%. Accordingly, it was concluded that there should be a minimum of 28 patients in a group. Figure 1 shows the CONSORT flowchart of the study. A post hoc power analysis showed that the study was reliable with a power and moderate effect of > 84%.

###  Inclusion criteria

1. Systemically healthy individuals, 18‒69 years of age
2. Nonvital mandibular premolar and molar teeth without periapical lesions and clinical symptoms
3. Asymptomatic nonvital teeth with a negative response to electrical and cold tests and a negative response to percussion and palpation tests
4. Premolar and molar teeth with a diameter of 2‒5 mm (PAI: 3‒4) of the periapical lesion without any calcification and obliteration in the pulp chamber on radiographic examination
5. Teeth without fractures, cracks, or apical resorption

###  Exclusion criteria

1. Patients that used drugs for pain and infection control, such as analgesics, anti-inflammatory agents, and antibiotics in the previous 12 hours
2. Patients with a history of susceptibility or adverse reactions to any drugs or materials used in the study
3. Presence of calcified root canals or severe periodontal problems in the tooth in question
4. Missing opposite or adjacent teeth
5. Pregnant or breastfeeding women

###  Randomization and blinding

 Eighty-nine patients were randomly assigned to three groups and blinded to the study group allocation. The randomization protocol was implemented at https://www.randomizer.org, a free resource for researchers to generate random numbers or randomly assign participants to experimental conditions. The patients were assigned to study groups according to the number sequence obtained on the site.

 For blinding purposes, sealed numbered syringes containing the final irrigation solutions were covered with identical tape, prepared, and packaged by a dental nurse not involved in the study so that the syringe contents were not visible and could not be distinguished. Freshly prepared solutions were matched with the group number for each patient and noted on the syringe. A dental assistant gave each solution to the dentist according to the patient’s group number. Therefore, neither the patient nor the physician knew which solution was applied. In addition, the statistician did not have any information about the characteristics of the groups during the statistical evaluation phase of the study. The groups were statistically analyzed using group numbers only.

###  Root canal treatment procedures

 All the root canal treatments were completed in a single session by one endodontist (SIY) with nine years of experience. Inferior alveolar nerve local anesthesia was applied using a 27-gauge, 2-inch dental needle (Set Injector; Set Medical Instruments, Istanbul, Turkey) with 1.5 mL of 40-mg/mL articaine and 0.01-mg/mL epinephrine (Maxicaine Forte, Aventis, Bridgewater, NJ, USA). A conventional root canal access cavity was prepared using round diamond burs (Merkez, Ankara, Turkey) under rubber dam isolation. The working length was determined with a #15 K-file (Dentsply, Sirona, Ballaigues, Switzerland) using the Root ZX apex locater (J. Morita Co, Tustin, CA) and checked with a diagnostic radiograph. Root canals were instrumented with ProTaper Next (PTN, Dentsply, Sirona, Switzerland) rotary instruments with a torque-controlled endomotor (SybronEndo, Orange, CA, USA). The apical diameters of the mesial roots were 25.06, and the distal root was 30.06. A lubricant (Glyde File Prep, Dentsply-DeTrey, GmbH, Konstanz, Germany) was used between each file to prevent rotary files from getting stuck in the root canal. During the instrumentation, 2 mL of 2.5% NaOCl (Werax, Izmir, Turkey) was applied to each root canal for 30 sec using a 30-gauge side-vented endodontic injector (Dentsply, Sirona, Switzerland), 1 mm shorter than the working length.

 After the irrigation protocol was completed using NaOCl, the final irrigation solutions were applied with a 30-gauge side-vented endodontic injector (Dentsply), 1 mm shorter than the working length, using the following protocols:

- Group 1 (Qmix, n = 29): After applying 2 mL of saline solution to each root canal for 30 sec, 2 mL of Qmix solution was applied for 60 seconds.
- Group 2 (EDTA, n = 30): After applying 2 mL of saline solution to each root canal for 30 sec, 2 mL of 17% EDTA was applied for 60 seconds.
- Group 3 (CHX, n = 30): After applying 2 mL of saline solution to each root canal for 30 sec, 2 mL of 2% CHX was applied for 60 seconds.

After all the irrigation procedures were completed, the root canals were dried using sterile paper points (DiaDent, DiaDent International, Burbany, BC, Canada). Next, the root canals were obturated with a single cone technique using gutta-percha (Dentsply, Sirona) suitable for master apical file and a root canal paste (AH Plus, Dentsply, Sirona) with epoxy resin content. After obturation, root canal filling was checked by radiography. Permanent restorations of the teeth were made with composite material (3M Espe, Turkey), and the patients were prescribed 100 mg of flurbiprofen to use when there was unbearable pain. All occlusal contacts were avoided following root canal treatment to prevent inappropriate traumatic occlusion.

###  Evaluation of postoperative pain

 Postoperative pain was evaluated using the verbal rating scale (VRS). Following the endodontic treatment, each patient was given a form to record their pain level. The patient was informed about the treatment procedure and the use of the form. Postoperative pain was recorded using a 4-level VRS 12, 24, 48, and 72 hours and one week after treatment. The patients were contacted at each time interval to remind them to record their pain level. The tooth types were also compared to each other (premolar and molar teeth). Pain levels were categorized as follows:

1. No pain; tooth feels normal
2. Mild pain that does not require taking analgesics
3. Moderate pain that diminishes with an analgesic
4. Severe pain that does not decrease with analgesics

###  Statistical analysis

 To maintain statistical blindness, the data obtained in this study were encoded into a Microsoft Excel (2010) spreadsheet (Microsoft Office, Redmond, CA). The statistician did not have any information about the working groups. SPSS 21 (SPSS Inc., Chicago, IL, USA) was used for statistical evaluation. Categorical variables were analyzed using Pearson’s chi-squared test. In addition, Fisher’s exact test and chi-squared analysis with Monte Carlo simulation were used. The significance level was set at 0.05 for all the tests.

## Results

 Of 266 patients referring to the clinic between October 2019 and February 2020, 92 met the inclusion criteria and agreed to participate in the study. Three patients were lost during the follow-up period. Therefore, 89 patients were statistically evaluated (Figure 1). No flare-up or swelling was observed in any patient during the follow-up period.

 Of 89 patients included in the study, 42 (47%) were female, and 47 (53%) were male. There was no statistically significant difference between gender and experimental groups (*P* = 0.434). The ages of the patients ranged from 20 to 69, with a mean age of 36.19 ± 13.77 years. A significantly higher pain level was observed in the EDTA group at 12 hours in patients over 60 years of age (*P* = 0.008, Table 1).

**Table 1:** Baseline characteristics of the included study participants in the Qmix, EDTA, andCHXgroups

**Variables**	**Qmix (n/%)**	**EDTA (n/%)**	**CHX (n/%)**	***P*** ** value**
Ages				
20-39	12 (41.3%)	14 (46.6%)	13 (43.3%)	0.008
40-59	11 (37.9%)	10 (33.3%)	11 (36.6%)	
60+	6 (20.6%)	6 (20%)*	6 (20%)	
Gender				
Female	16 (55.1%)	12 (40%)	17 (56.6%)	0.434
Male	13 (44.9%)	18 (60%)	13 (43.4%)	
Tooth				
Mandibular premolar	4 (13.7%)	7* (23.3%)	5 (16.6%)	0.012
Mandibular molar	25 (86.3 %)	23 (76.7 %)	25 (83.3%)	

*P < 0.05

 Postoperative pain distribution among the groups, according to time intervals, is shown in Table 2. The incidence of moderate postoperative pain at 12 hours in the CHX group (26.1%, *P* = 0.751) was higher than that in the other groups. Although the group with the highest “no pain” ratio was the Qmix group (62.1%), there were no statistically significant differences between the groups at 12 hours (*P* = 0.751). At 24 hours, the highest incidence of “mild pain” was observed in the EDTA group at 33.3%. CHX and EDTA resulted in a higher pain severity than Qmix (*P* = 0.019). There were no significant differences between groups in postoperative pain values at 48- and 72-hour and 1-week intervals (*P* > 0.05). At 72 hours in the Qmix group, 100% of the patients reported “no pain” postoperatively. At the 1-week interval in the EDTA group, 100% reported “no pain” postoperatively. However, at the 1-week interval in the CHX group, “mild pain” was still reported (6.7%).

**Table 2:** A Comparison of pain levels in terms of the final irrigation solutions

**Pain levels**	**Qmix (n/%)**	**EDTA (n/%)**	**CHX (n/%)**	***P*** ** value**
12 hours				0.751
None	18 (62.1%)	14 (46.7%)	14 (46.7%)	
Mild	6 (20.7%)	9 (30%)	7 (23.3%)	
Moderate	5 (17.2%)	7 (23.3%)	8 (26.7%)	
Severe	0 (0%)	0 (0%)	1 (3.3%)	
24 hours				
None	27 (93.1%)	20 (66.7%)	21 (70%)	0.019*
Mild	2 (6.9%)	10 (33.7%)	7 (23.3%)	
Moderate	0 (0%)	0 (0%)	2 (6.7%)	
Severe	0 (0%)	0 (0%)	0 (0%)	
48 hours				
None	27 (93.1%)	26 (86.7%)	24 (80%)	0,375
Mild	2 (6.9%)	4 (13.3%)	6 (20%)	
Moderate	0 (0%)	0 (0%)	0 (0%)	
Severe	0 (0%)	0 (0%)	0 (0%)	
72 hours				
None	29 (100%)	28 (93.3%)	27 (90%)	0,362
Mild	0 (0%)	2 (6.7%)	3 (10%)	
Moderate	0 (0%)	0 (0%)	0 (0%)	
Severe	0 (0%)	0 (0%)	0 (0%)	
1 week				
None	29 (100%)	30 (100%)	28 (93.3%)	0.333
Mild	0 (0%)	0 (0%)	2 (6.7%)	
Moderate	0 (0%)	0 (0%)	0 (0%)	
Severe	0 (0%)	0 (0%)	0 (0%)	

* *P* < 0.05

 When comparing pain levels between groups by tooth type, the incidence of postoperative pain in premolar teeth (18,8 %) was significantly higher than in molar teeth at 72 hours (*P* = 0.012). At 72 hours in the EDTA group, reports of mild pain in premolar teeth were statistically higher than in other groups (*P* = 0.012). No significant differences in pain level were observed between tooth types and groups at other time intervals (*P* = 0.054) (Table 2). There were no statistically significant differences in the analgesics used between the groups. While 92.8% of patients took analgesics between 0 and 12 hours, 7.2% of patients took analgesics between 12 and 24 hours. Patients did not take any analgesics at 48-hour, 72-hour, and 1-week intervals.

## Discussion

 Postoperative pain following root canal treatment negatively affects patients’ quality of life.^17^ Previous studies have compared postoperative pain and irrigation solutions used during root canal treatment.^16,18^ This study was designed to investigate the effects of various final irrigation solutions on postoperative pain following root canal treatment.

 In the present study, all the treatment procedures were performed in a single session to avoid possible factors that might cause pain, such as intracanal medication or leakage of temporary fillings. The working length was detected with an electronic apex locater and confirmed by radiographs. The Root ZX apex locater was used to determine the working length because of its accuracy.^19^ A rotary system was used in all the patients, following the manufacturer’s instructions.

 Due to its low biocompatibility and incidence of toxic effects on periradicular tissues,^12^the concentration of NaOCl used in this study was 2.5% to minimize toxic effects and benefit from antimicrobial properties. In the present study, 2.5% NaOCl, saline, and 17% EDTA were used sequentially in the EDTA group. Additionally, a saline solution was used to prevent brown toxic precipitates when CHX and NaOCl solutions were combined.

 Qmix, containing EDTA, CHX, and surfactants, such as detergents, has shown high antimicrobial properties. While the EDTA component of Qmix dissolves the smear layer, the CHX ingredient has strong antimicrobial properties.^13^As indicated by previous studies, no irrigation solution possesses all the desired properties.^7^The present study showed that the use of Qmix as a final irrigation after NaOCl positively affects and decreases postoperative pain in patients. This decrease is statistically significant at 24 hours. This positive effect of the Qmix solution might be related to its effective role in removing the smear layer^14^ and high antibacterial efficacy.^13^ In a study by Yılmaz et al,^16^ postoperative pain level after root canal treatment using Qmix with Endo Activator (EA, Dentsply, Sirona), was significantly lower compared to NaOCl. Yilmaz et al^16^ attributed the reduction in postoperative pain to the use of EA. Since there are no other clinical studies comparing Qmix as a final irrigation solution concerning postoperative pain following root canal treatment, it is not possible to compare the data. One study found Qmix more biocompatible than other irrigation solutions.^20^ Another study found Qmix more effective than NaOCl in biofilm removal.^21^The authors attributed this finding to the effective removal of the smear layer by the ingredients in Qmix. In the present study, patient pain level was significantly lower with Qmix than other groups, similar to previous studies.^16^Thus, the null hypothesis was rejected.

 Due to its wide antimicrobial effect and low cytotoxicity, making it effective in root canal irrigation and medication, CHX is used as an endodontic irrigation solution.^22^In a previous study conducted by Almeida et al,^23^no differences occurred in postoperative pain following root canal treatment between two groups treated with 5.25% NaOCl and 2% CHX. In another study, greater postoperative pain was reported when CHX was used as an intracanal medicament, compared to calcium hydroxide.^24^Contrary to these studies, Bashetty and Hegde^25^used 5.25% NaOCl and 2% CHX in nonvital teeth and the pain level was statistically lower in the first 6 hours with CHX. In the present study, more severe pain was reported with CHX versus Qmix at 24 hours. Although CHX is a biocompatible solution, Qmix is thought to have higher biocompatibility due to its components.^14^In addition, more severe postoperative pain at 24 hours with EDTA, compared to Qmix and CHX, might be due to the detergent structure of EDTA, resulting in the exacerbation of an existing inflammatory state.

 Preoperative pulpal and periapical status are important factors in postoperative pain.^26^In the current study, only nonvital mandibular posterior teeth (premolar and molar) were used. In previous studies, more severe postoperative pain was reported in molar teeth compared to premolar and anterior teeth.^27^In addition, the complex anatomy of mandibular molar teeth affects cleaning of the lateral canals in the apical region of the root canal and healing of periapical lesions. Therefore, mandibular molars with necrotic pulp allow the evaluation of the worst case scenario.^28^In the present study, while there were no statistically significant differences between premolar and molar teeth and groups at 12, 24, and 48 hours, there was a statistically higher pain level in the EDTA group at 72 hours in molar teeth. However, in the present study, the number of premolar teeth was lower compared to molar teeth. Therefore, additional studies with an equal number of premolar and molar teeth are recommended.

 In the current study, postoperative pain severity was evaluated using VRS. It was categorized as no pain, mild, moderate, and severe.^29^The VRS scale allows for identification of pain and quantification with numerical data. In addition, the visual analog scale (VAS) is a valid and reliable method for pain measurement. The VAS scale exactly predicts pain, not by range, but by ratio. However, pain is influenced by various factors; therefore, the VRS scale, with only 4 categories, was used to facilitate the rating process for the patient.

 Patients in this study were instructed to use analgesics as needed. These instructions were given to prevent psychological anxiety related to pain and prevent patient anxiety from influencing the results of the study.

 The patients were randomized prior to treatment. Randomization reduces bias in treatments. Various randomization methods have been used in clinical studies to prevent bias between the operator and patients (flipping a coin, choosing colored balls in a bag, choosing cards, etc.).^16,30^In the present study, randomization was performed regularly, the operator did not interfere with the allocation of patients to any treatment group, and the preoperative status of patients in each group was similar.

## Conclusion

 Qmix caused less pain levels in 24 hours than other solutions. There was no difference between irrigation solutions after one week. Qmix was associated with less severe postoperative pain, and it can be concluded that using Qmix as a final irrigation solution in endodontic treatment is appropriate.

## Acknowledgements

 The authors thank Dr Karakaş for statistical analysis.

## Authors’ Contributions

 SIY contributed to the study concept and design, acquisition of data, drafting of the manuscript, and critical revision. KO performed the analysis and interpretation of data and critical revision.

## Funding

 None.

## Ethics Approval

 The study design was approved by the Ethics Committee of Istanbul Medipol University (No: 929). And ClinicalTrials.gov. with the Ref No. NCT04310254.

## Competing Interests

 The authors deny any conflict of interest.
